# Herz und Herz-Kreislauf-System in der (kultur-)historischen Betrachtung

**DOI:** 10.1007/s00059-020-04914-2

**Published:** 2020-04-14

**Authors:** Ulrich Koehler, Olaf Hildebrandt, Wulf Hildebrandt, Gerhard Aumüller

**Affiliations:** 1 Klinik für Innere Medizin, SP Pneumologie, Intensiv- und Schlafmedizin, Universitätsklinikum Giessen und Marburg GmbH, Philipps-Universität Marburg, Baldingerstraße 1, 35033 Marburg, Deutschland; 2 Medizinische Zellbiologie, Institut für Anatomie und Zellbiologie, Philipps-Universität Marburg, Marburg, Deutschland; 3 Emil-von-Behring-Bibliothek, Arbeitsstelle für Geschichte der Medizin, Philipps-Universität Marburg, Marburg, Deutschland

**Keywords:** Blutzirkulation, Medizingeschichte, Kulturgeschichte, Erkenntnisgewinn, Wissenschaftlicher Fortschritt, Blood circulation, Medicine history, Cultural history, Gain of knowledge, Scientific progress

## Abstract

Wer waren die Entdecker des Herz-Kreislauf- und des Kapillarsystems? Fragt man die Studenten eines höheren Semesters nach historischen Bewandtnissen, die den Weg der Medizin in die Gegenwart entscheidend beeinflusst haben, so gibt es in der Regel keine oder eine falsche Antwort. Wer wissenschaftliches Denken und Handeln erfahren will, kommt nicht umhin, sich auch mit der historischen und kulturellen Entwicklung der Medizin auseinanderzusetzen. Die Naturwissenschaften und die Medizin liefern jedoch wie kaum eine andere Wissenschaft den Beweis, dass im Sinne der Patienten beständig nach neuen Wegen und Erkenntnissen gesucht werden muss. Dieser Artikel soll einen Beitrag dazu leisten, daran zu erinnern, wie es zur Entdeckung des Herz-Kreislauf-Systems und der kulturhistorischen Bedeutung des Herzens gekommen ist. Nicht zuletzt soll er jedoch auch einen Eindruck vermitteln, unter welch großem Einsatz – mitunter des eigenen Lebens – und auf welch steinigem Wege der Erkenntnisgewinn zustande gekommen ist.

## Abstract (en)

Who discovered the cardiovascular and capillary systems? When students in advanced semesters are asked about historical matters that have decisively influenced the path to present day medicine, as a rule no answer or a false answer is forthcoming. Whoever wants to understand scientific thinking and action, cannot do better than to grapple with the historical and cultural developments in medicine; however, more than any other science the natural sciences and medicine provide evidence that new ways and knowledge must be consistently sought for the benefit of patients. The aim of this article is to make a contribution to remembering how the cardiovascular system was discovered and the cultural and historical importance of the heart. Last but not least, however, the article aims to convey the impression of the huge personal sacrifice, including one’s own life, and the stony path which led to the acquisition of this knowledge.

## Einleitung

Mit dem Herzen sind bis heute magische und mystische Vorstellungen verbunden. Das Herz ist für den Menschen seit jeher mehr als nur ein Körperorgan; es wird sinnbildlich für den ganzen Menschen und seine Seele gesehen. Kaum ein Wort bzw. Symbol wurde und wird so oft benutzt wie das des Herzens. Das Herz als Sitz der Seele gilt gleichzeitig als Symbol der Liebe und des Lebens. Liebe und Leben bleiben in der Poesie noch immer mit dem klopfenden Herzen verbunden. Das regelmäßige Schlagen des Herzens hatte schon für die Ägypter etwas Faszinierendes, der Puls galt als „Sprache des Herzens“. Das Herz wusste alles und musste bei der großen Abrechnung nach dem Tode vor dem Totenrichter als Zeuge auftreten. Auch mit der Beschreibung der Anatomie und Funktion des Herzens durch den Engländer William Harvey (1628) wurde das Herz seiner mystischen und magischen Phantasien nicht beraubt. Die Entdeckung des Blutkreislaufs durch Harvey hatte für die Medizin insofern eine enorme Bedeutung, als erstmalig Herz-Kreislauf-Erkrankungen sowie deren Interaktion mit anderen Organsystemen erkannt werden konnten. Im 18. und 19. Jahrhundert hat man sich weiterhin intensiv mit der Anatomie und Physiologie des Herzens auseinandergesetzt. Die Morphologie der Herzklappen, das Erregungsleitungssystem sowie die Anatomie der Herzkranzgefäße und die Bestimmung der Drucke und Volumina in Vorhöfen und Kammern wurden mittlerweile bis ins Detail erforscht und beschrieben. Heute wissen wir, dass das Herz-Kreislauf-System ein komplex geregeltes Feedbacksystem darstellt, das in Abhängigkeit von unterschiedlichen Aktivitätsgraden eine optimale Blutversorgung und Perfusion aller Organsysteme gewährleistet.

## Das Herz in der Hochkultur der Ägypter (3100–300 v. Chr.)

Die zentrale Rolle des Herzens über Jahrtausende hinweg spiegelt sich v. a. in seiner besonderen Behandlung bei der Bestattung von Pharaonen, Kaisern und Reichen wider. Für die Ägypter waren irdisches Leben und Tod lediglich Zwischenstationen auf dem Weg zum ewigen Leben. Im Jenseits gab es ein Weiterleben auf Ewigkeit, das aber nur möglich war, wenn die körperliche Hülle eines verstorbenen Menschen unversehrt und funktionsfähig blieb. Um den Körper eines Toten für die Ewigkeit zu erhalten und vor dem Verfall zu schützen, sahen die Ägypter die Notwendigkeit, diesen zu öffnen und zu konservieren. Für die Ägypter war das Herz das Lebenszentrum und der Sitz des Denkens und Fühlens. Bevor die Seele ihren Weg ins Jenseits antreten konnte, wurde sie einer Prüfung vor dem Totengericht unterzogen. Das Totengericht mit dem obersten Richter, dem Totengott Anubis, hatte über die Lebensführung eines Menschen sowie über seine guten und schlechten Taten zu befinden. Man ging davon aus, dass das Herz vor dem Totengericht als unbestechlicher Zeuge über den Verstorbenen aussagen würde. Anubis hatte das Herz auf der Seelenwaage zu wiegen, um festzustellen, ob der Verstorbene rechtschaffen gelebt hat. Hatte er die Wahrheit gesagt und die Prüfung bestanden, bewegte sich die Waage nicht. Die Seele konnte sich nun wieder mit dem Körper zu einer Einheit verbinden. Senkte sich die Waagschale, war für den Verstorbenen ein Weiterleben im Jenseits unmöglich geworden. Viele Papyri und Grabbeilagen enthalten Hinweise darauf, dass das Herz beim Totengericht positiv für den Verstorbenen aussagen sollte. Der Tod eines Menschen war für die Ägypter nur eine kurzzeitige Phase der Trennung von Körper und Seele. Mit der Balsamierung des Körpers sowie dem Begräbniszeremoniell mit Wiederbelebungsritus und der Prüfung vor dem Totengericht war die Einheit von Körper und Seele wiederhergestellt. Beim Balsamierungsprozess wurde das Herz in eine eigens dafür vorgesehene Kanope gelegt, später hat man es im Körper belassen. Oftmals wurde das Herz auch durch einen „Herzskarabäus“, das Sinnbild der Regeneration, ersetzt und dieser in den Brustkorb der balsamierten Leiche gelegt. Die zentrale Stellung des Herzens in der ägyptischen Kultur hat die hebräische, griechische und römische Philosophie, Religion und Kultur stark beeinflusst. Die später im christlichen Abendland propagierte Herzbestattung ist sicherlich auch auf die herausragende Stellung des Herzens in der ägyptischen Kultur zurückzuführen.

Dass Herzschlag und Puls in Zusammenhang stehen, wurde schon im Papyrus Ebers um 1550 v. Chr. beschrieben. Von den Papyri ist der Papyrus Ebers mit einer Länge von 20 Metern die umfangreichste Quelle zur ägyptischen Medizin. Er wurde 1862 von dem Amerikaner Edwin Smith in Luxor im Antikenhandel erworben und 1873 an den Leipziger Ägyptologen Georg Ebers veräußert. Der Papyrus enthält unter anderem auch Abhandlungen über die Bedeutung des Herzens und der Gefäße. Die Ärzte der Pharaonenzeit wussten bereits von der Beziehung des Herzens zum Pulsschlag, von der Funktion des Herzens als Pumpe und von den Kanälen der zu den unterschiedlichen Körperteilen führenden Gefäße. Die Ägypter haben die zentrale Stellung des Herzens im Gefäßsystem bereits erkannt. Der Schlag des Herzens führt dazu, dass jede Körperstelle mit Lebensstoffen (Luft und Wasser) versorgt werden kann. Zitat aus dem Papyrus Ebers: „Das Herz spricht vorn/außen in den Gefäßen einer jeden Körperstelle“. In getrennten Gefäßen werden Luft und Wasser zu den Organen transportiert. Pathophysiologisch ist das Gefäßsystem damit auch in der Lage, Krankheitsstoffe an alle Orte des Körpers zu tragen. Kommt es zu Stockungen der Ausscheidung, bilden sich Krankheiten. Die Existenz einer zentralen Pumpe (Herz) und eines Röhrensystems zum Transport lebensnotwendiger Stoffe ist pathophysiologisch sehr bedeutsam. Das Kanal- oder Röhrensystem des Körpers, vergleichbar einem Bewässerungssystem der Felder, belegt bereits ein sehr früh existierendes anatomisches Ordnungsprinzip der Ägypter.

## Das Herz in der Schöpfungsgeschichte

Über 800-mal kommt das Wort „Herz“ in den Bibelversen vor. Das Herz ist in der Bibel das Zentrum menschlichen Seins, gute und schlechte Charaktereigenschaften des Menschen werden mithilfe des Herzens versinnbildlicht. Mit dem Herzen verbinden sich Zuneigung und Abneigung, Liebe und Hass, Treue und Untreue sowie Freude und Traurigkeit. Das Herz kann redlich und willig, es kann aber auch feige, arglistig und verstockt sein. „Glückselig, die reinen Herzens sind, denn sie werden Gott sehen.“

## Vertreter der alexandrinischen Schule

Herz und Leber, die im Streit um den Sitz der Seele lange Zeit Rivalen gewesen sind, mussten sich gegen das von Hippokrates favorisierte Gehirn als Seelenort verteidigen. Das Corpus hippocraticum ging von 2 Herzkammern aus. Man mutmaßte, dass die rechte Herzkammer und die Venen Blut enthielten, während linke Herzkammer und Arterien mit Luft gefüllt seien (aerterein = Luft enthalten). Aristoteles (384–322 v. Chr.), Philosoph und Biologe, hat das Herz als allmächtiges Organ und Sitz der Seele angesehen. Aristoteles war davon überzeugt, dass Herzerkrankungen unvereinbar mit dem Leben seien. Seiner Auffassung nach konnte eine Seele nur in einem warmen Organ wie dem Herzen wohnhaft sein. Zum Leben gehört Wärme. Das Gehirn war für Aristoteles nichts weiter als das Kühlsystem des Herzens. Aristoteles beschrieb 3 Herzkammern, den Puls sah er als Resultat der Aufwallung des Blutes an. Der Anatom Herophilos von Chalkedon (etwa 280 v. Chr.) und der Physiologe Erasistratos von Keos (etwa 250 v. Chr.) haben wesentliche Erkenntnisse zur Anatomie der Blutgefäße, des Transports von Blut und Pneuma sowie zur Funktion des Herzens und der Herzklappen beigetragen. Herophilos von Chalkedon unterschied zwischen Arterien und Venen. Er war der Auffassung, dass der Puls durch die Gefäße selbst erzeugt würde. Zudem könne man anhand des Pulses Rückschlüsse auf den Gesundheitszustand eines Menschen ziehen. Für Erasistratos von Keos, Assistent von Herophilos, war das Herz Ausgangspunkt der Flüssigkeits- und Pneumabewegung, wobei das Pneuma das Lebensprinzip darstellte.

Galen von Pergamon (129–ca. 210), der bedeutendste Arzt des klassischen Altertums, war Gladiatorenarzt in seiner Heimatstadt Pergamon, bevor er nach Rom ging. Er hat auf die Entwicklung der Medizin über viele Jahrhunderte hinweg großen Einfluss ausgeübt. Bis zum 17. Jahrhundert blieb der „Galenismus“ die unumstößliche medizinische Lehre. Galen befasste sich neben der praktischen ärztlichen Tätigkeit v. a. mit Anatomie und Physiologie. Er führte Vivisektionen an Tieren durch und gewann dadurch elementare Einblicke in die Anatomie des tierischen Körpers, transportierte damit zugleich aber auch einige Fehler in die menschliche Anatomie. Galen lehrte in Rom, dass das Herz der „Ort des inneren Feuers und der Entstehung des spiritus vitalis“ sei. Blut wurde als Nährstoff des Körpers oder im Falle von Krankheit als Quelle von Entzündungen und Fieber gesehen. Das durch Galen vervollkommnete Krankheitskonzept der hippokratischen Säftelehre hat das Fundament ärztlichen Erkennens und Handelns bis in die frühe Neuzeit bestimmt.

**Figure 1:**
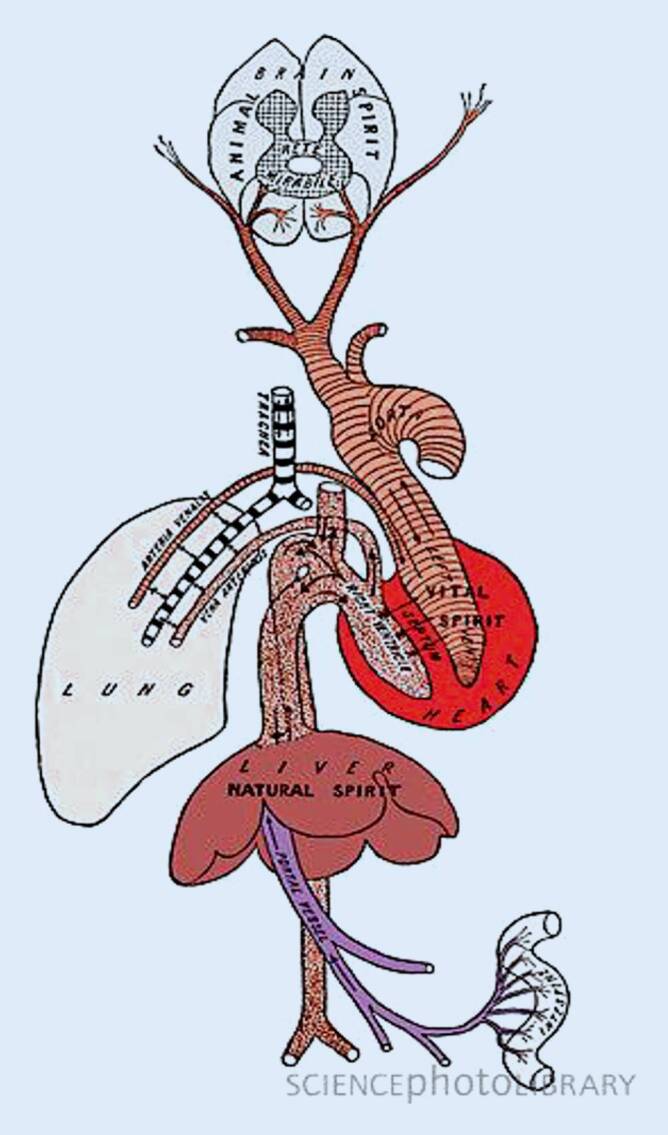

Bezüglich der Blutentstehung sowie der Blutbewegung hat Galen eine eigene Theorie entwickelt. Er hat das zweigekammerte Herz als eine Art Saugpumpe verstanden. Die Leber war der Ort kontinuierlicher Blutproduktion, sie wurde mit Nahrung aus Magen und Darm versorgt. Aus der Leber gelangt das Blut (mit dem „spiritus naturalis“) in die rechte Herzkammer. In der rechten Herzkammer bilden sich 2 Blutströme – der eine geht in die Lunge, der andere durch Poren in der Herzscheidewand in die linke Herzkammer (Abb. 1). Der Grundstoff des Lebenspneumas, die Luft, wird von der Lunge in die linke Herzkammer gesaugt und dort, unter Vermittlung des inneren Feuers, in das Lebenspneuma „spiritus vitalis“ umgewandelt. Von der linken Herzkammer wird das angereicherte Blut zu den Organen und dem Gehirn transportiert. Dort wird es komplett verbraucht, ein Kreislauf war für Galen nicht existent. Die bei der Bildung des Lebenspneumas entstandenen rauchigen Verbrennungsprodukte werden wiederum retrograd über die Lunge ausgeschieden. Das Lebenspneuma liefert gleichzeitig auch den Grundstoff für das im Hirn gebildete Seelenpneuma. Der entscheidende Punkt in Galens Beschreibung des Herz-Gefäß-Systems ist, dass er „Poren“ in der Herzscheidewand annimmt, durch die Blut vom rechten in das linke Herz gelangt. Demnach würde nur ein geringer Teil des Blutes vom rechten Ventrikel in die Lunge fließen, gerade ausreichend, um diese zu ernähren. Es besteht nach diesem spekulativen Konzept also kein Kreislauf, sondern das Blut wird in Systole und Diastole wie Ebbe und Flut im Körper bewegt, und die Organe entnehmen ihm je nach Bedarf ihren Anteil an „spiritus vitalis“.

## Ibn al-Nafis al Quarashi (1210–1288)

Im 13. Jahrhundert entdeckte Ibn al-Nafis als Erster, dass das Blut in einem Kreislauf durch die Lunge fließt. Ibn al-Nafis war ein syrischer Universalgelehrter, der nicht nur auf dem Gebiet der Medizin, sondern auch auf dem der Theologie, Philosophie, Astronomie und der Rechtswissenschaft aktiv war. Die anatomischen und physiologischen Vorstellungen, die er niedergeschrieben und gezeichnet hat, sind vermutlich erst sehr viel später in den europäischen Raum gelangt. Im Alter von 29 Jahren veröffentlichte er seine wichtigste Arbeit, einen Kommentar zur Anatomie in Avicennas „Canon“. Ibn al-Nafis hat seine Vorstellungen nicht auf anatomische Erfahrungen durch Sektionen stützen können, sondern allein auf theoretische Überlegungen. Er hat als Erster die Lungenpassage bzw. den kleinen Kreislauf beschrieben. Seiner Auffassung zufolge floss das Blut durch die rechte Herzkammer und die Lungenarterie hindurch in die Lunge, wurde dort angereichert und anschließend über die Lungenvene ins linke Herz geleitet. Ibn al-Nafis widerspricht der Lehrmeinung Galens, dass es Poren für den Blutdurchtritt in der Herzscheidewand gäbe.

## Leonardo da Vinci (1452–1519)

**Figure 2:**
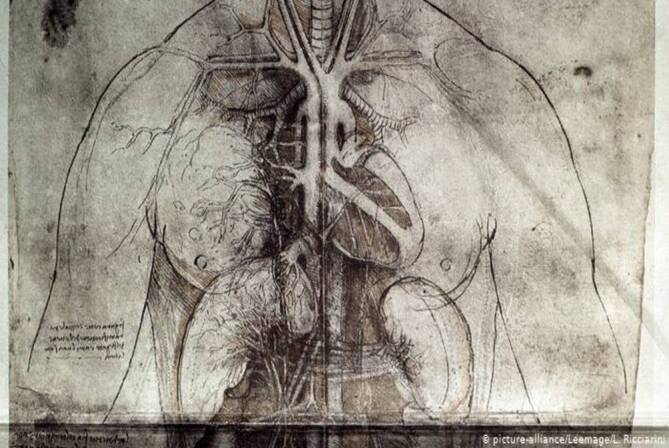

**Figure 3:**
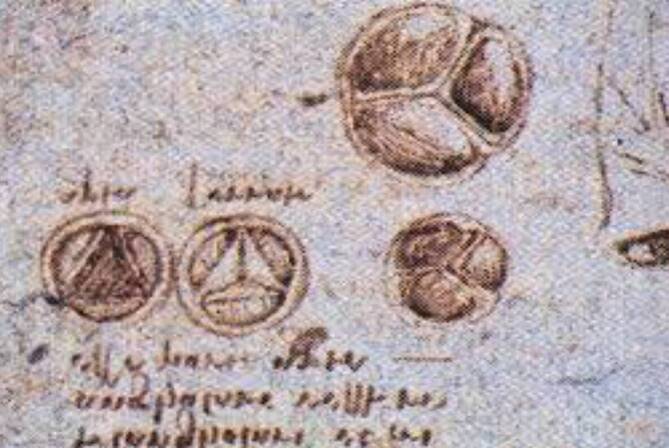

Der Italiener da Vinci war ein Universalgenie – er war zugleich Wissenschaftler, Ingenieur und Künstler. Leonardo hat in Florenz Sektionen an Leichen durchgeführt und eine Vielzahl anatomischer Zeichnungen von hervorragender künstlerischer Qualität und Anschaulichkeit angefertigt (Abb. 2). Durch seine Faszination für die Strömungstechnik hat er Entdeckungen zum Fluss des Blutes an der Aortenklappe gemacht, die erst in den letzten Jahrzehnten wissenschaftlich bestätigt und gewürdigt worden sind. Leonardo beschreibt einen spiralförmigen Blutfluss durch die Teile der Aorta, die heute als Sinus Valsalvae bekannt sind. Der Analyse der Strömungsverhältnisse und des Blutflusses an der Aortenklappe sowie deren Schließmechanismus hat Leonardo etliche Seiten mit Zeichnungen und Erklärungen gewidmet (Abb. 3). Bis Mitte des 20. Jahrhunderts ging man davon aus, dass die Aortenklappe sich durch das in der Aorta retrograd gestaute Blut schließt (Druckumkehr). Anhand von Experimenten hat Leonardo jedoch nachgewiesen, dass der Blutstrom spiralartig in die Aorta hineinfließt und es davon abhängig zu einem fluiddynamischen Schluss der Klappe kommt. In den 1960er-Jahren konnte dieser Sachverhalt wissenschaftlich bestätigt werden. Die Aortenklappe funktioniert nicht im Sinne eines Rückflussventils. Man hat erkannt, dass die Blutflusswirbel in der Aortenwurzel einen Druck auf das Segel sowie die Sinus ausüben und demzufolge die Schließung des Segels allmählich und synchronisiert entsteht. Die heutigen Anatomen bezeugen, dass Leonardo eine verblüffend präzise Darstellung der Blutflussverhältnisse in der Aortenwurzel geliefert hat. Leonardo korrigiert die seit Galen bestehende Vorstellung, dass das Herz nur 2 Herzkammern habe. Er beschreibt Herzkammern und Vorhöfe. Er bestätigt Galens falsche Vorstellungen, dass das Herz zur Erwärmung des Blutes notwendig sei und dass die Kammern über Poren des Septums miteinander kommunizieren. Leonardo hat das Herz als Druckpumpe verstanden, auf die Idee eines Blutkreislaufs ist er nicht gekommen. Sein Skizzenbuch ist erst am Ende des 19. Jahrhunderts zum Druck gekommen, die Originale waren über etwa 200 Jahre unter Verschluss. Leonardo ist in Frankreich gestorben; nach seinem Tode wurden die Skizzenbücher seinem Freund Francsco Melzi vermacht.

## Michael Servetus (1511–1553) und Realdo Colombo (1516–1559)

1553 hat Servetus, ohne Kenntnis des Schrifttums von Ibn al-Nafis, ebenfalls einen die Lunge passierenden Kreislauf beschrieben. Servetus war ein spanischer Arzt, Theologe und Rechtswissenschaftler. Er hat, abweichend von den Theorien Galens, festgestellt, dass die Vermischung des Blutes mit der Atemluft in der Lunge erfolge und dieses dann den Weg in die linke Herzkammer nehme. Nach Servetus’ Auffassung gab es auch keine Poren in der Herzscheidewand, durch die das Blut hätte fließen können. Seine von der Kirchenlehre abweichenden Ansichten zur Trinität machten ihn zu einem religiös Verfolgten. Am 26. Oktober 1953 wurde er vom Schweizer Reformator Calvin der Gotteslästerei für schuldig befunden und in Genf auf dem Scheiterhaufen öffentlich verbrannt. Der Italiener Realdo Colombo, Schüler von Vesalius, bestätigt Servetus’ Beschreibung des Lungenkreislaufs. Colombos physiologische Beobachtungen basierten primär auf Vivisektionen von Tieren.

## Andreas Vesalius (1514–1564)

Die Entdeckungsreisen der Anatomen und Pathologen in das Körperinnere förderten viele neue Erkenntnisse zu Tage. Im 14. und 15. Jahrhundert waren Sektionen häufiger geworden, ihr Erkenntnisgewinn blieb aber vergleichsweise gering. Bei diesen ging es hauptsächlich darum, Lehrmeinungen zu bestätigen, insbesondere diejenigen von Galen. Im 16. Jahrhundert beeinflussten die Renaissance sowie die Philosophie die anatomische Lehrmeinung. Der berühmte Anatom Andreas Vesalius, der im Alter von 23 Jahren zum Professor der Anatomie an die Universität Padua berufen wurde, zweifelte bereits früh an der Richtigkeit der Lehrsätze von Galen. Im Jahre 1543 veröffentlichte er sein Hauptwerk über die Anatomie („De humani corporis fabrica libri septem“, 7 Bücher über den Bau des menschlichen Körpers). Vesalius hat durch eine Vielzahl von Sektionen Unterschiedlichkeiten zwischen den dogmatisch gelehrten klassisch-anatomischen Vorstellungen Galens und den selbst gewonnenen Erkenntnissen feststellen können. Viele Fehler der Anatomie Galens waren unter anderem der falschen Analogie der Tier- zur Menschenanatomie geschuldet. Die Anatomie von Galen wurde mit Vesalius’ anatomischen Studien nicht überwunden, es ist allenfalls zu Korrekturen gekommen. Bei aller an Galen geübten Kritik ist es Vesalius vermutlich aber auch mehr darum gegangen, Galens Werk zu vollenden, zumal er seine aufrichtige Bewunderung für ihn nie verleugnet hat. Seitens der Ärzteschaft und v. a. seines Lehrers Sylvius wurde den neuen Theorien von Vesalius mit Ablehnung und Unverständnis begegnet. Er musste den Lehrstuhl in Padua verlassen.

## Johannes Dryander (1500–1560)

**Figure 4:**
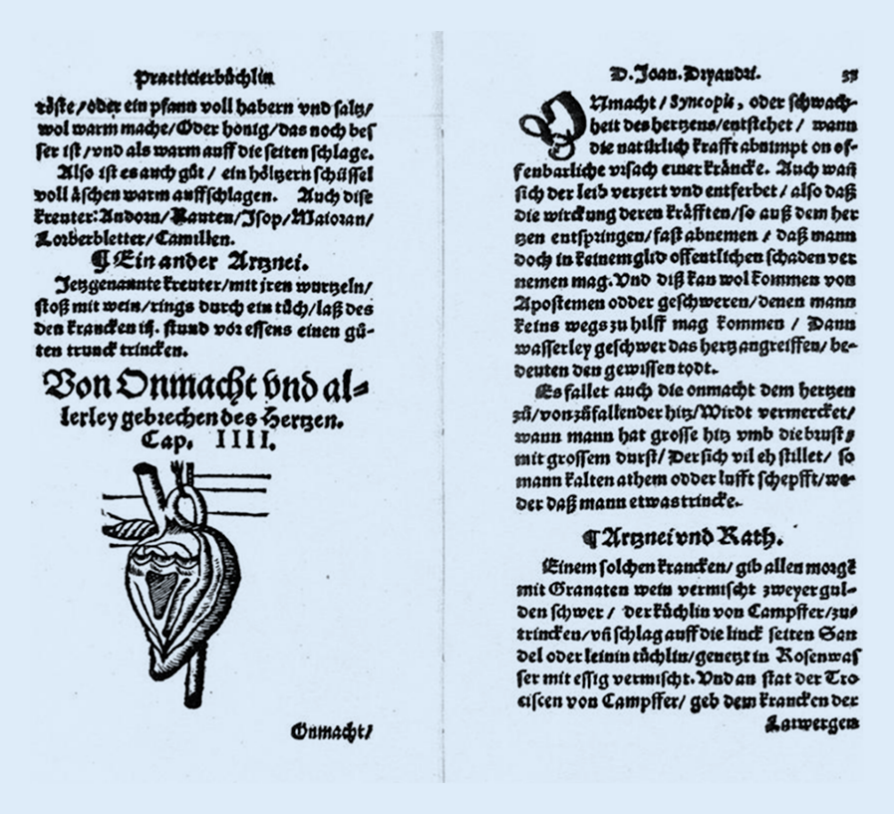

Eine ähnliche Vorstellung wie bei Vesal lässt sich auch aus der Abbildung ableiten, die der Marburger Anatom, Mathematiker, Geo- und Kosmograph und Leibarzt der hessischen Landgrafen, Johannes Dryander (1500–1560, gräzisiert aus Eichmann), in seinem 1557 erschienenen „New Artznei vnd Practicirbüchlin/zu allen Leibs gebrechen vnd Kranckheyten/[…]“ dem 3. Kapitel beigegeben hat. Dieses handelt „Von Onmacht vnd allerley gebrechen des Hertzen“ und zeigt ein isoliertes Herz, bei dem offenbar die rechte Kammer eröffnet ist und die Taschenklappen der Lungenschlagader zu sehen sind (Abb. 4). Ihm quasi angehängt, erscheint der Aortenbogen, der hakenartig von einem dünnen Strang umschlungen wird; vermutlich ist hier der Nervus vagus mit dem N. recurrens gemeint. Ähnlich fehlerhaft wie die großen Gefäße sind auch die Herzinnenräume dargestellt. Das rechte Herzohr erscheint als abgespreiztes flügelartiges Gebilde ohne Verbindung zum Innenraum des Herzens, bei dem die Herzhöhle des rechten Ventrikels als dreieckiger Hohlraum mit strangartigen Untergliederungen, vermutlich den Segelklappen mit den Chordae tendineae, angedeutet ist und der linke Ventrikel nur einen Spaltraum in der nach unten sehr zugespitzt dargestellten Masse des linken Herzens darstellt.

Dryander hatte zu Beginn seiner Tätigkeit in Marburg ab 1535 mehrere öffentliche Sektionen durchgeführt, mit die ersten an einer deutschen Universität, und konnte daher auf eigene Beobachtungen zurückgreifen. Weit weniger kritisch als sein genialer Zeitgenosse Andreas Vesalius (1514–1564) hat er sich dabei für die Abbildungen seiner „Anatomiae, hoc est corporis humani dissectionis pars prior“ (Marburg 1537) offenbar weniger auf seine eigenen Beobachtungen als vielmehr auf die Beschreibungen früherer Autoren, z. B. des berühmten Mundinus (Raimondo dei Luzzi, Bologna, um 1275–1326) verlassen. So geht er nicht auf die von Galen geforderten „Poren“ im Ventrikelseptum ein (die aber auch Vesal geflissentlich übergeht).

Ähnlich traditionell beschreibt Dryander in seinem „Practicirbüchlin“ dann auch die Erkrankungen des Herzens und ihre Behandlungen mit kühlenden Medikamenten wie Granatapfelwein, mit Kampfer vermischt, oder essighaltiges Rosenwasser. Den Grund für diese (auf der antiken Viersäftelehre beruhenden) Behandlung leitet er aus einer Beschreibung ab, die vielleicht als eine frühe Äußerung über stenokardiale Beschwerden verstanden werden kann: „Es fallet auch die onmacht dem hertzen zu/von zufallender hitz/Wirdt vermercket/wann mann hat grosse hitz vmb die brust/mit großem Durst/Der sich vil eh stillet/so mann kalten athem odder lufft schepft/weder daß mann etwas trincke.“

## Das Herz als Opfergabe bei den Azteken (14.–16. Jahrhundert n. Chr.)

Seit undenklichen Zeiten hat das Wissen um die Macht der Götter, Ahnen und Dämonen einen großen Teil des menschlichen individuellen und gesellschaftlichen Handelns bestimmt. Die Menschen haben im Glauben gelebt, dass Götter und Dämonen dazu befähigt wären, den Lauf des Lebens zu beeinflussen. Demzufolge hielt man es für notwendig, die Götter gnädig zu stimmen. Die Azteken als Hochkultur Mittelamerikas hatten ein Herzritual, das besonders grausam war. Grundlage dieses Rituals war der Glaube, die den Menschen Leben spendende Sonne benötige Herzen und Blut als Nahrung. So wurden Tausende Kriegsgefangene, die man die steilen Stufen der Tempeltreppen hinauf zu den Priestern gebracht hatte, in einem bestialischen Ritual getötet. Mit steinernen Messern wurden den Gefangenen die Herzen bei lebendigem Leib aus dem Körper herausgeschnitten und den Göttern geopfert. Die verstümmelten Körper der Toten wurden die Pyramidentreppen hinabgestürzt und zum Verzehr freigegeben. Die Azteken lokalisierten den Sitz des Willens im Herzen. Sie hatten Furcht, dass die am Abend untergehende Sonne am nächsten Morgen nicht mehr aufgehen könnte. Die Verbindung zwischen Sonne und Herz war für die Azteken etwas ganz Besonderes. Insofern kam dem Herzopfer kein Strafcharakter zu. Ganz im Gegenteil: Die Opferung war als göttlicher Tod mit Botschaft zu verstehen.

## William Harvey (1578–1657)

**Figure 5:**
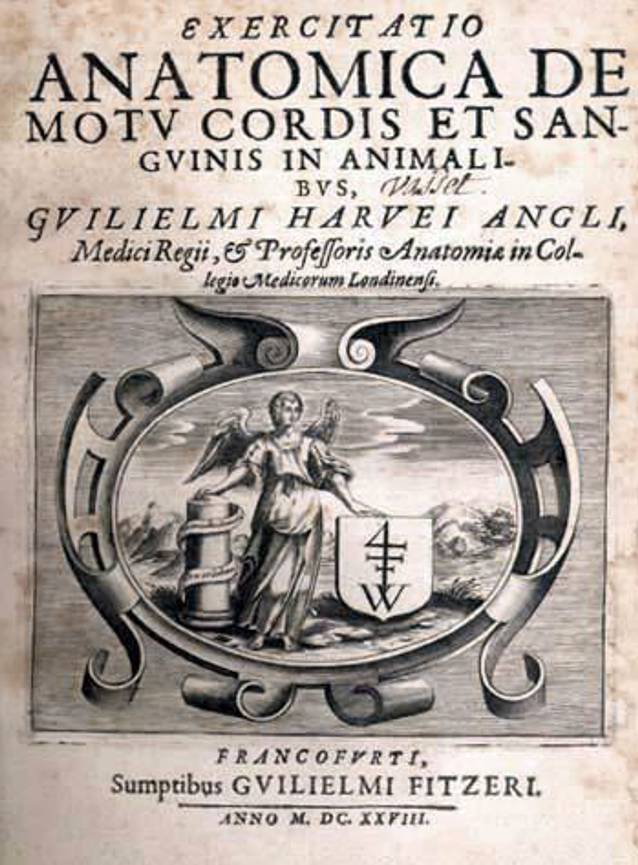

William Harvey sieht das Herz als „Urquell des Lebens“, das dazu benötigt wird, das Blut zu erwärmen und ihm „seine Vollkommenheit“ wiederzugeben. Harvey, Sohn eines Kaufmanns, begann das Studium der Medizin an der Universität in Cambridge und ging dann mehrere Jahre an die Universität nach Padua. Sein dortiger Lehrer, der berühmte Anatom Fabricius ab Aquapendente (1537–1619), hat in erheblichem Maße dazu beigetragen, dass sich Harvey für die Blutzirkulationstheorie begeistert hat. Fabricius hat sich in Padua insbesondere um die Bedeutung der Venenklappen bemüht. Schon während seiner Studien in Italien begann Harvey Untersuchungen zur Funktionsweise des Herzens. Bereits 1603 war er sich in der Vorstellung ziemlich sicher, dass die Bewegung des Blutes konstant auf kreisförmige Art und Weise erfolge und das Ergebnis des Herzschlags sei. Das Herz funktioniere wie ein Muskel, die Herzkammern würfen in der systolischen Kontraktion Blut aus. Die Arterien pulsierten aufgrund der Druckwelle des schlagenden Herzens und nicht durch eine eigene Pulsationskraft. Zurück in London, widmete sich Harvey neben der Praxis, der Tätigkeit am Hospital und seinen Funktionen beim Royal College of Physicians v. a. der Anatomie und der Physiologie. Harveys Argumente beruhten auf anatomischen Sektionen, experimenteller Physiologie, mathematischer Berechnung und der Beobachtung. Er hat von der Undurchdringlichkeit der Herzscheidewand durch Vesal gewusst, ebenso vermutlich von der Lungenpassage des Blutes durch Servetus und Colombo. Die von Galen propagierte Verbindung zwischen rechter und linker Herzkammer, die „Galen-Poren“, war für ihn nicht existent. Rechtes und linkes Herz waren anatomisch getrennt, das rechte Herz pumpte das Blut in die Lunge und das linke pumpte es in den Körper. Der Mut, sich von der antiken Blutbewegungstheorie von Galen zu verabschieden und sich ein neues Konzept anhand eigener physiologisch-morphologischer Beobachtungen auszudenken, stellt die eigentliche Leistung von William Harvey dar. Mathematische Berechnungen der vom Herzen in das geschlossene Röhrensystem der Gefäße abgegebenen Blutmenge, Vivisektionen von Tieren sowie experimentelle Untersuchungen haben zum Beweis der Zirkulation beigetragen. Die Unerschrockenheit, eine durchaus kritische Distanz zur Lehrmeinung sowie der Glaube an die „wahren Philosophen, die von Wahrheitsliebe und Weisheitsliebe brennen“ belegen Harveys unerschütterlichen Willen und seine Motivation zur Veröffentlichung der Ergebnisse seiner jahrelangen Untersuchungen. Aus seinem berühmten, 1628 in Frankfurt am Main publizierten Werk „Exercitatio anatomica de motu cordis et sanguinis in animalibus“ seien einige Sequenzen zitiert, die uns in eindrücklicher Art und Weise Harveys Vorstellung von der Zirkulation verdeutlichen (Abb. 5):

## Zitat 7. Kapitel

In der Leber steckt nichts, was einen Anstoß gibt, keine Triebkraft, in der Lunge wird durch den Schlag der rechten Herzkammer das Blut hineingedrängt, und durch dessen Anstoß müssen die Gefäße und Poren der Lunge erweitert werden, wie dies bei Badeschwämmen geschieht und bei jeglichen Gebilden, die einen schwammigen Bau haben, sobald sie zusammengepresst werden und sich wieder erweitern.

## Zitat 8. Kapitel

So dürfte es wahrscheinlich auch im Körper zustande kommen, dass alle Teile durch die Blutbewegung mittels nährkräftigen Blutes genährt, durchwärmt und belebt werden, dass das Blut hingegen in den Körperteilen abgekühlt, verdichtet und geschwächt wird, daher es zu seinem Ursprung, und zwar zum Herzen, gleichsam zu seiner Quelle bzw. zum Hausaltar des Körpers zurückkehrt, um seine Vollkommenheit wieder zu erlangen. So ist das Herz der Urquell des Lebens und die Sonne der „kleinen“ Welt, so wie die Sonne im gleichen Verhältnis den Namen Herz der Welt verdient.

## Zitate 15. Kapitel

Denn ein jedes Gemütsleiden, das die menschlichen Seelen mit Lust und Schmerz, Hoffnung oder Ängstlichkeit erregt, dringt sowohl bis zum Herzen, und dies erzeugt dort auch eine Umwandlung der natürlichen Beschaffenheit im Wärmezustand, im Puls und im Übrigen, indem es von Anbeginn den gesamten Nährstoff verdirbt und die Kräfte schwächt.

Da alle Lebewesen von innerlich verkochter Nahrung leben, ist es notwendig, daß die Verkochung und die Verteilung vollkommen sind und dass daher ein Ort und Behältnis vorhanden ist, wo die Nahrung fertiggestellt wird und woher sie in die einzelnen Glieder abgeleitet wird. Dieser Ort ist das Herz …

Harvey hat die Blutzirkulation im Körper erstmalig vollständig beschrieben. Seiner Theorie zufolge war das Herz ein Transportorgan, welches zudem der Erwärmung und Auffrischung des Blutes diente. Seit der Antike besteht die wichtigste Funktion des Herzens in der Produktion und Regelung von Wärme. Dies ist der entscheidende Prozess, der wiederum alle anderen Funktionen des Körpers steuert und energetisch unterhält. Das Herz ist die zentrale Heizung des Körpers. Im Unterschied zu Galen brennt im Herzen keine Flamme mehr, deren rauchartige Rückstände über die Lunge an die Außenwelt abgeatmet werden müssten. Die „Verkochung der Nahrung“ und die Anreicherung des Blutes mit energiehaltigem Nährstoff waren nach Harveys Vorstellung eine wesentliche Funktion des Herzens. Alle Teile des Körpers wurden durch die Blutbewegung mittels nährkräftigen Blutes versorgt, durchwärmt und belebt. Das aus den Körperteilen kommende „abgekühlte, verdichtete und geschwächte Blut“ musste zum Herzen zurückkehren, um dort wieder vervollkommnet zu werden. Harvey sieht das Herz somit als Zentralorgan des Lebens: das Herz als Sonne, das die Lebensvorgänge überhaupt erst möglich macht. Die eigentliche Funktion der Lunge ist Harvey erstaunlicherweise noch nicht bekannt. In der zweiten Antwort an seinen Widersacher, den Pariser Anatomen Jean Riolan, ist er sich sicher, dass keine gasförmigen Bestandteile aus der Luft aufgenommen werden. Bei der Ausatmung werde das Blut von Schlacken bereinigt, bei der Einatmung werde es gekühlt.

## Marcello Malpighi (1628–1694)

**Figure 6:**
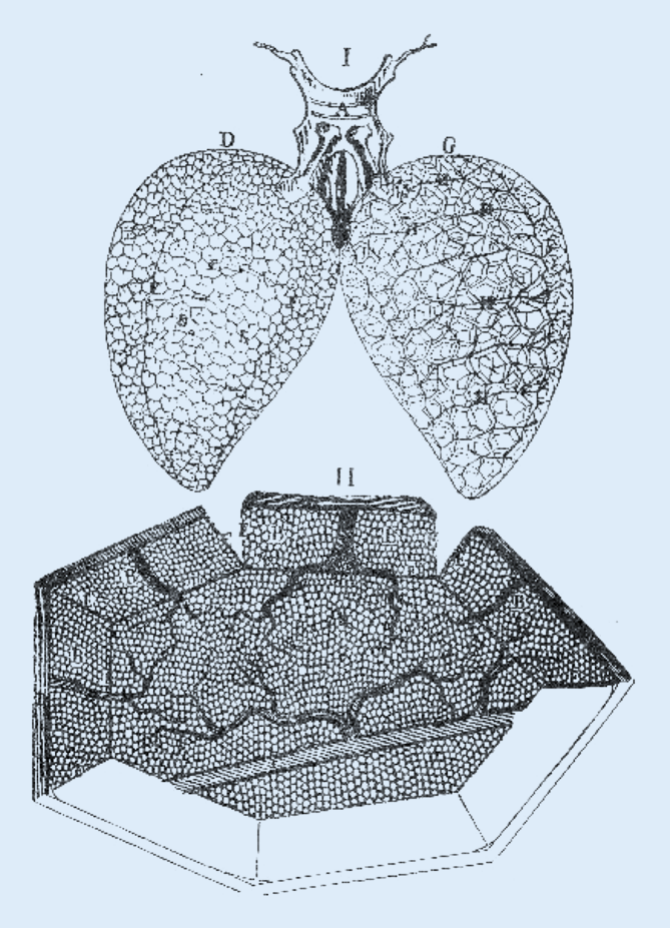

Das, was wir heute als physiologisch selbstverständlich ansehen, die Zirkulation, hat Jahrhunderte in der Bestätigung gebraucht. Die apodiktische Lehrmeinung einer unantastbaren Autorität, wie der des Galen von Pergamon, hat über Jahrhunderte hinweg dazu beigetragen, neue Erkenntnisse zu verhindern. Endgültig anerkannt wurde die „Zirkulationstheorie“ Harveys durch den Nachweis des Kapillarsystems der Lunge durch Marcello Malpighi (Abb. 6). Der Blutübertritt von der Arterie über die Kapillaren in die Vene hat den Kreislauf als geschlossenes System bestätigt. Erst mit der Einführung des Mikroskops in der zweiten Hälfte des 17. Jahrhunderts konnte noch das letzte Puzzleteil ergänzt werden. Malpighi, Begründer der mikroskopischen Anatomie, ist mit der Entdeckung des pulmonalen Kapillarsystems auch dem Verständnis des Gasaustausches sehr nahegekommen.

## Herzbestattungen im europäischen Adel (16. und 17. Jahrhundert)

Die Sehnsucht des Menschen, über den Tod hinaus einen Platz unter den Lebenden in der Heimat zu behalten, führte bei den Mächtigen des Abendlandes zu der Sitte, Körper und Herz an unterschiedlichen Stätten begraben oder aufbewahrt zu wissen. Die Herzbestattung war bei den Mächtigen und Reichen über viele Jahre hinweg üblich, insbesondere im 16. und 17. Jahrhundert. Dieser Brauch verdeutlicht in eindrücklicher Art und Weise, dass man dem Herzen, dessen Tod früher mit dem leiblichen Tod gleichgesetzt wurde, eine besondere Bedeutung beigemessen hat. Die Herzen mancher Verstorbenen wurden sogar als Reliquien bei den Hinterbliebenen aufbewahrt. Auch Richard Löwenherz, der legendäre König von England, ließ seine sterblichen Überreste auf drei heilige Stätten verteilen, um so nach seinem Tod überall gegenwärtig zu sein. Die zentrale Bedeutung des Herzens spiegelt sich somit in der besonderen Behandlung bei der Bestattung von Pharaonen, Kaisern und Königen sowie Mächtigen und Reichen wider: Die Konservierung des Herzens unter der Vorstellung ewigen Lebens und der Auferstehung. Ein Beispiel für die Übertragung dieses Rituals in die bürgerliche Welt ist die im Museum anatomicum Marburg aufbewahrte Silberbüchse mit dem trocken präparierten Herz des Begründers der Sammlung, des Anatomen Christian Heinrich Bünger (1781–1842), die die Aufschrift trägt: „Dieses edle Herz schlug zum Wohle der leidenden Menschheit“.
